# Maternal use of a combination of recreational and antiretroviral drugs (nyaope/whoonga): Case reports of their effects on the respiratory system in infants

**DOI:** 10.7196/AJTCCM.2021.v27i3.112

**Published:** 2021-10-04

**Authors:** C P Mashiloane, P H Jeena, S A Thula, S A Singh, R Masekela

**Affiliations:** Paediatric Intensive Care Unit, Inkosi Albert Luthuli Central Hospital, Durban, South Africa

**Keywords:** nyaope, whoonga, Illicit drugs, respiratory adverse effects, maternal drug use, airway obstruction

## Abstract

Nyaope/whoonga is an indigenous street drug in South Africa (SA). It is made from a combination of neuro-stimulatory illicit drugs such as antiretroviral drugs, heroin, cannabis, opioids, cocaine as well as common household powders such as flat-screen television glass powder. It is a very addictive substance and is used even during pregnancy. Its effects on the developing fetus have been described as causing neonatal abstinence syndrome (NAS), intrauterine growth restriction (IUGR) and neurological complications. There are no data in the literature that report its effect on the respiratory system (RS) of the fetus or neonates. We describe two children who were prenatally exposed to nyaope and presented with upper and lower respiratory tract obstructions associated with recurrent pneumonias. Further studies are required to describe the adverse effects of whoonga on the developing RS of prenatally exposed fetuses.

## Case 1

A seven-month-old girl was admitted to Inkosi Albert Luthuli Central Hospital, Durban, for upper airway obstruction (UAO) and severe pneumonia with moderate acute respiratory distress syndrome (ARDS). Her background history revealed that she had an intrauterine growth restriction (IUGR). She was born to an HIV-positive, 23-year-old mother who was a dagga, nyaope, tobacco and alcohol addict. The grandmother was the primary caregiver because the mother had a history of aggression and child neglect. The baby had been previously admitted for moderate acute malnutrition. Although at high risk of exposure to HIV, her HIV PCR test was negative. She had previously required invasive ventilation three times for UAO.

Clinically, she was noted to have subtle dysmorphism (up-slanting palpebral fissures, hypertelorism and macroglossia). She was jittery and had features of autonomic instability, i.e. unexplained tachycardia which was thought to be secondary to persistent withdrawal symptoms. She had stridor and respiratory distress requiring ventilation. Her examination of the airway under anaesthesia by the otolaryngologist, was unremarkable (no subglottic stenosis nor laryngomalacia) except for upper airway oedema. The vocal cords had normal movement. She was also treated for pneumonia with antibiotics. During this stay in the paediatric intensive care unit (PICU), parainfluenza 3, adenovirus and herpes simplex virus were isolated from the endotracheal aspirates.

**Fig. 1:**
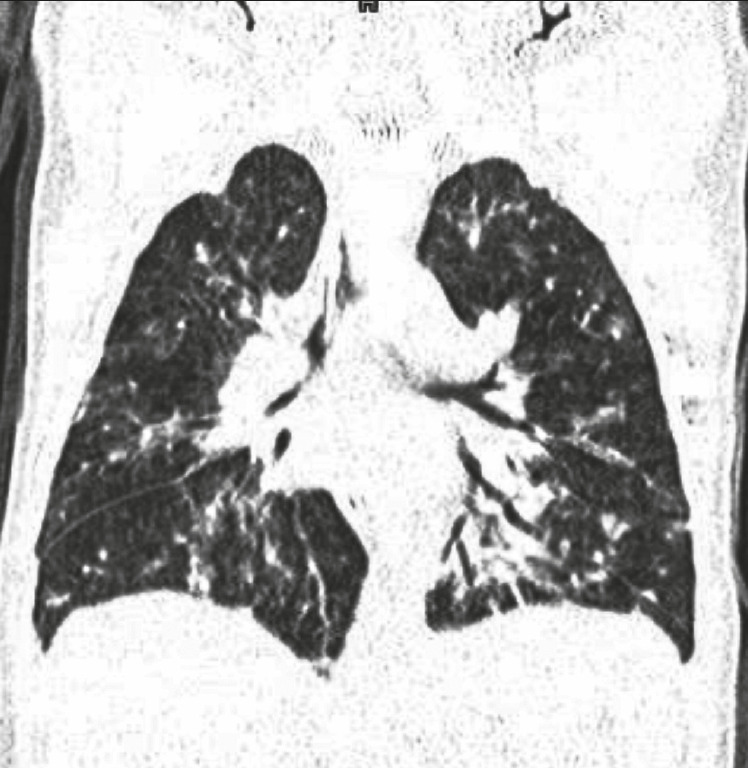
Bilateral perihilar consolidation with features of bronchiolitis and chronic lung disease.

She had recurrent and prolonged PICU admissions (a total of 6 months) with multiple failed extubations and inability to wean off the ventilator because of persistent UAO and lower airway obstruction (LAO) refractory to intravenous (IV) bronchodilators. Therefore a tracheostomy was performed. A high-resolution computed tomography (CT) scan of the chest was done to exclude bronchiolitis obliterans (BO) as the cause of persistent lower airway obstruction. The results showed features in keeping with bronchiolitis (diffuse centrilobular nodules with ground glass appearance and air-trapping); however, there were no features of BO identified (Fig. 1). Sadly, after a prolonged stay in PICU and ventilator dependence, she died owing to overwhelming sepsis, septic shock and multi-organ dysfunction.

## Case 2

A nine-month-old boy admitted to the PICU for UAO and pneumonia with ARDS. He had a background history of being born prematurely at 35/40 weeks’ gestation and had IUGR. His mother was 20 years old and HIV-negative and known to be addicted to nyaope with use during pregnancy and postnatally. He had previously been admitted to the neonatal intensive care unit (NICU) and was intubated for congenital pneumonia and NAS requiring midazolam and morphine infusions.

He presented with severe UAO, i.e. subglottic stenosis, which required emergency tracheostomy. He also had superimposed pneumonia. Clinically, he was not dysmorphic and other systems were normal. Cardiac echography was normal. While admitted to the PICU, he developed PICU-related complications which included polymicrobial nosocomial pneumonias with sepsis and septic shock, episodes of blocked tracheostomy with prolonged hypoxia and associated air leak syndrome.

He developed refractory LAO requiring IV bronchodilators and was also ventilator dependent. At this point there was a suspicion of tracheomalacia or bronchomalacia. A flexible bronchoscopy was performed and only revealed a right main bronchus plug. He remained oxygen dependent with persistent LAO. After spending a total of 73 days in PICU with 64 days on the ventilator, he was discharged from the PICU. The mother was noted to care poorly for the child while admitted in a general ward and therefore was planned for admission to a rehabilitation centre. The child was released into the care of the paternal family on discharge.

## Discussion

These two cases illustrate the variable forms of abnormal respiratory pathology. Both cases had *in utero* exposure to illicit drugs and presented with severe UAO requiring tracheostomy. They later developed severe recurrent pneumonias with persistent LAO requiring intravenous bronchodilators, ventilator dependence for prolonged periods and eventually developed chronic lung disease.

Whoonga, also known as nyaope, is an illegal concoction indigenous to South Africa (SA).^[1,2]^ It consists of heroin, methamphetamine, cannabis, cocaine and common household powders such as rat poison, ammonia, chlorine, flat screen television glass powder and antiretroviral drugs, i.e. efavirenz or ritonavir. One can become addicted from first use and it has extremely severe withdrawal symptoms.^[2]^ The uniqueness of the drug lies in its demographic popularity exclusively among African people.^[3]^ Poverty is associated with the use of illegal drugs.^[4]^ In SA, illegal drugs such as cannabis, whoonga and cocaine are commonly used among women including pregnant women.^[1]^ Whoonga has not been widely studied specifically for its effects on the respiratory system (RS). Thomas *et al*.^[3]^ reported two neonatal cases with neurological complications.

Postnatal effects of other illicit drugs used during pregnancy are clearly documented and these include IUGR, prematurity, low birthweight and neonatal abstinence syndrome (NAS).^[5,6]^ Other reported adverse effects include gastroschisis, neural tube defects, congenital cardiovascular disease, and long-term neurological effects on these children. ^[7]^

There is a paucity of information on what respiratory effects nyaope has on the developing lung. However, when the nyaope ingredients are dissected and explored individually, the literature has documented effects of some of the individual drugs on the RS. Tashkin *et al*. ^[8]^ reported that cocaine causes bronchial constriction and is associated with worsening asthma.^[8]^ Cannabis is associated with a risk of developing airway disease and lung cancer.^[9]^ Withdrawal and stress-related behaviours are often seen in neonates exposed to cannabis, as it increases carboxyhaemoglobin levels, impairs oxygen transfer in the lung and reduces oxygen-carrying capacity of blood.^[9]^ A pooled analysis of eight birth cohorts showed maternal smoking in pregnancy is associated with likelihood of wheezing and asthma in children.^[10]^

In both our cases, the mothers also abused tobacco and alcohol. Maternal genotype influences the risk for low birthweight and pulmonary function in the offspring of cigarette smokers.^[11]^ The cytochrome P450 family 1, subfamily A, polypeptide 1(CYP1A1), glutathione S-transferase Mu 1 (GSTM1) and glutathione S-transferase theta 1(GSTT1) genes encode proteins that eliminate toxic substances contained in cigarette smoke. Maternal smoking was found to be associated with decreased lung function in children whose mothers had CYP1A1a/aa and GSTM1 absent genotype.^[11]^

Experimental studies of the effects of nicotine on lung development in mice showed thicker alveolar walls, increased airway smooth muscle and collagen deposition, airway hyper-responsiveness with airflow restriction and airway growth affectation.^[12,13]^

Alcohol is well known for inducing fetal alcohol syndrome and may play a role in sudden infant death syndrome when there is prenatal exposure. A study on rats reported that prenatal exposure to ethanol reduces breathing frequency, impairs response to hypoxia and reverses long-term facilitation of respiration.^[14]^ Exposure to opiates such as heroin is associated with NAS, developmental delay and behavioural problems.^[7]^ Moreover, opiate exposure is also associated with respiratory depression in neonates.^[7]^ Efavirenz has been found to cause anencephaly, anophthalmia and cleft palate in monkeys;^[15]^ however, there are no reports suggesting it has respiratory effects on the exposed developing fetus.

If these individual drugs have such effects on the RS, we presume that when combined they may have even worse detrimental AEs on the developing RS.

Tracheostomy care is challenging, and these children require a safe environment for adequate care. We observed complications of tracheostomy while admitted in PICU and both children had extremely poor social circumstances. The risk for unplanned readmission of children with tracheostomy is 2.6 times higher in families with substance abuse and the risk of respiratory infections was doubled in cases of household cigarette smoke exposure.^[16]^

## Conclusion

These two cases report similar respiratory complications in children who were prenatally exposed to nyaope. Illicit drugs use during pregnancy is associated with many AEs on the fetus and the neonate. Other systemic sequelae of these drugs have not been explored, particularly their impact on the developing RS of the exposed fetus. Further studies on the effects of this dangerous drug cocktail are warranted.
